# Supplementary material for: Prevalence, associated factors, and association of intimate partner violence and suicidal behaviors among women of reproductive age in Asia: Protocol for a systematic review and meta-analysis of cross-sectional studies
Source: PLoS One. 2026 Apr 10;21(4):e0338752. doi: 10.1371/journal.pone.0338752 (PMC13068250; doi:10.1371/journal.pone.0338752)
Supplement: S1 Table — (DOCX) [file pone.0338752.s001.docx]

**1. Search Strategy for PubMed**

| **Search Element** | **Search Terms and Structure** |
| --- | --- |
| **Outcome (O)** | ("Intimate Partner Violence*"[MeSH] OR "Domestic Violence"[MeSH] OR "Spouse Abuse"[MeSH] OR "intimate partner violence"[Title/Abstract] OR "domestic violence"[Title/Abstract] OR "spousal abuse"[Title/Abstract] OR "partner violence"[Title/Abstract] OR "partner abuse"[Title/Abstract] OR "spouse abuse"[Title/Abstract] OR "wife beating"[Title/Abstract] OR "battered woman"[Title/Abstract]) |
|  | ("Suicide"[MeSH] OR "Suicidal behaviour*"[MeSH] OR "Suicide, Attempted"[MeSH] OR "Self-Injurious Behavior"[MeSH] OR "suicidal ideation"[Title/Abstract] OR "suicide ideation"[Title/Abstract] OR "suicide attempt"[Title/Abstract] OR "attempted suicide"[Title/Abstract] OR "self-harm"[Title/Abstract] OR "self-harm"[Title/Abstract] OR "self-injury"[Title/Abstract] OR "self injur*"[Title/Abstract]) |
|  | ("risk factors"[Title/Abstract] OR "associated factors"[Title/Abstract] OR "determinants"[Title/Abstract] OR "predictors"[Title/Abstract] OR "correlates"[Title/Abstract] OR "contributors"[Title/Abstract] OR "protective factors"[Title/Abstract] OR "modifiable factors"[Title/Abstract] OR "non-modifiable factors"[Title/Abstract]) |
| **Population (P)** | ("Female"[MeSH] OR female*[Title/Abstract] OR woman[Title/Abstract] OR women[Title/Abstract] OR "women of reproductive age"[Title/Abstract] OR "reproductive-age women"[Title/Abstract] OR "reproductive age"[Title/Abstract] OR "15-49"[Title/Abstract]) |
| **Location** | ("Asia*"[MeSH] OR Asia[Title/Abstract] OR "South Asia*"[Title/Abstract] OR "Southeast Asia*"[Title/Abstract] OR "East Asia*"[Title/Abstract] OR "Western Asia*"[Title/Abstract] OR "Central Asia*"[Title/Abstract] OR Afghanistan[Title/Abstract] OR Bangladesh[Title/Abstract] OR Bhutan[Title/Abstract] OR India[Title/Abstract] OR Pakistan[Title/Abstract] OR Nepal[Title/Abstract] OR "Sri Lanka"[Title/Abstract] OR Maldives[Title/Abstract] OR China[Title/Abstract] OR Japan[Title/Abstract] OR Korea[Title/Abstract] OR "South Korea"[Title/Abstract] OR "North Korea"[Title/Abstract] OR Mongolia[Title/Abstract] OR Taiwan[Title/Abstract] OR Kazakhstan[Title/Abstract] OR Kyrgyzstan[Title/Abstract] OR Tajikistan[Title/Abstract] OR Turkmenistan[Title/Abstract] OR Uzbekistan[Title/Abstract] OR Indonesia[Title/Abstract] OR Malaysia[Title/Abstract] OR Philippines[Title/Abstract] OR Singapore[Title/Abstract] OR Thailand[Title/Abstract] OR Vietnam[Title/Abstract] OR Laos[Title/Abstract] OR Cambodia[Title/Abstract] OR Myanmar[Title/Abstract] OR Brunei[Title/Abstract] OR "Timor-Leste"[Title/Abstract]) |

**2. Search Strategy for Scopus and PsycINFO**

| **Search Element** | **Scopus Search Terms** | **PsycINFO Search Terms** |
| --- | --- | --- |
| **Outcome (IPV)** | (TITLE-ABS-KEY("intimate partner violence" OR "domestic violence" OR "spousal abuse" OR "partner violence" OR "partner abuse" OR "spouse abuse" OR "wife beating" OR "battered woman")) | (DE "Domestic Violence" OR DE "Spouse Abuse" OR TI,AB,KW("intimate partner violence" OR "domestic violence" OR "spousal abuse" OR "partner violence" OR "partner abuse" OR "spouse abuse" OR "wife beating" OR "battered woman")) |
| **Outcome (Suicidal Behaviors)** | (TITLE-ABS-KEY("suicidal behaviour*" OR "suicidal behavior*" OR "suicidal ideation" OR "suicide ideation" OR "suicide attempt" OR "attempted suicide" OR "self-harm" OR "self harm" OR "self-injury" OR "self injury" OR "self-injur*")) | (DE "Suicide" OR DE "Suicidal Ideation" OR DE "Suicide Attempts" OR DE "Self-Injurious Behavior" OR TI,AB,KW("suicidal ideation" OR "suicide attempt" OR "attempted suicide" OR "self-harm" OR "self injury" OR "self-injur*")) |
| **Risk Factors** | (TITLE-ABS-KEY("risk factors" OR "associated factors" OR determinants OR predictors OR correlates OR "protective factors" OR "modifiable factors" OR "non-modifiable factors")) | (TI,AB,KW("risk factors" OR "associated factors" OR determinants OR predictors OR correlates OR "protective factors" OR "modifiable factors" OR "non-modifiable factors")) |
| **Population (Women)** | (TITLE-ABS-KEY(female* OR woman OR women OR "women of reproductive age" OR "reproductive-age women" OR "reproductive age" OR "15-49")) | (DE "Females" OR TI,AB,KW(female* OR woman OR women OR "women of reproductive age" OR "reproductive-age women" OR "reproductive age" OR "15-49")) |
| **Location (Asia)** | (TITLE-ABS-KEY(Asia OR "South Asia" OR "Southeast Asia" OR "East Asia" OR "Western Asia" OR "Central Asia" OR Afghanistan OR Bangladesh OR Bhutan OR India OR Pakistan OR Nepal OR "Sri Lanka" OR Maldives OR China OR Japan OR Korea OR "South Korea" OR "North Korea" OR Mongolia OR Taiwan OR Kazakhstan OR Kyrgyzstan OR Tajikistan OR Turkmenistan OR Uzbekistan OR Indonesia OR Malaysia OR Philippines OR Singapore OR Thailand OR Vietnam OR Laos OR Cambodia OR Myanmar OR Brunei OR "Timor-Leste")) | (TI,AB,KW(Asia OR "South Asia" OR "Southeast Asia" OR "East Asia" OR "Western Asia" OR "Central Asia" OR Afghanistan OR Bangladesh OR Bhutan OR India OR Pakistan OR Nepal OR "Sri Lanka" OR Maldives OR China OR Japan OR Korea OR "South Korea" OR "North Korea" OR Mongolia OR Taiwan OR Kazakhstan OR Kyrgyzstan OR Tajikistan OR Turkmenistan OR Uzbekistan OR Indonesia OR Malaysia OR Philippines OR Singapore OR Thailand OR Vietnam OR Laos OR Cambodia OR Myanmar OR Brunei OR "Timor-Leste")) |
