# Supplementary material for: Prevalence, associated factors, and association of intimate partner violence and suicidal behaviors among women of reproductive age in Asia: Protocol for a systematic review and meta-analysis of cross-sectional studies
Source: PLoS One. 2026 Apr 10;21(4):e0338752. doi: 10.1371/journal.pone.0338752 (PMC13068250; doi:10.1371/journal.pone.0338752)
Supplement: Attachment — Submitted filename: Response to Reviewers_PLOS-2026_R1.docx [file pone.0338752.s003.docx]

13^th^ March, 2026.

**Saeed Ahmad**

**Academic Editor**

**PLOS One**

**Subject: Revision submission for Manuscript (ID #PONE-D-25-62887)**

Dear Editor,

Thank you for informing me the decision regarding my manuscript. I sincerely appreciate the reviewers’ time and constructive comments. After carefully reviewing the feedback, we firmly hope and believe that it will substantially improve the overall quality of the manuscript. The point by point response of reviewer comments are attached below.

We hope the revised version would be strengthen scientifically and acceptable for publication in PLOS One. Thank you very much, and we are looking forward to hearing from you soon.

On behalf of all the authors,

**Dr. Mantaka Rahman** _(Corresponding author)_

icddr,b

Dhaka, Bangladesh.

**RESPONSES TO THE REVIEWER 1**

1. **Reviewer’s Comment:** Very interesting title: Despite it is current issues which need interventions, it is overlooked issues.

***Authors’ response:*** We thank the reviewer for his concern. The suggested point by point responses are attached below.

2. **Reviewer’s Comment:** Design and registration

• Line 151: please provide the link

***Authors’ response:*** The link of the registration has been addressed accordingly **(p6, Line 159).**

3. **Reviewer’s Comment:** Eligibility criteria

• Line 167: This is mixed method study and please use CoCoPo for prevalence which condition context and population, and PEO for exposure review and risk review population, exposure and outcome. compactor and s- study is not needed for this particular review. for study type you can describe under inclusion and exclusion criteria please cit PRISMA 2020 guideline

***Authors’ response:*** The suggested following changes have been incorporated accordingly. We have added CoCoPoP in the Table 1; incorporated PEO for the review by excluding comparator and study. Additionally, we have also cited PRISMA 2020 in Line 135 accordingly in the revised manuscript.

4. **Reviewer’s Comment:** Line 192: Table 1: Example Search Strategy for PubMed Database (PICOS Framework)

• This is mixed method study and please use CoCoPo for prevalence which condition context and population, and PEO for exposure review and risk review population, exposure and outcome. compactor and s- study is not needed for this particular review. for study type you can describe under inclusion and exclusion criteria please cit PRISMA 2020 guideline

***Authors’ response:*** The suggested following changes have been incorporated accordingly in the following section.

5. **Reviewer’s Comment:** Quality (risk of bias) assessment

• Line 196: RoB is not JBI tool. it is Cochran tool. so, which tool do you plan to use JBI or Cochrane?

• why only cross-sectional studies?

Data management

• line 197: Line 208: Do you mean study selection?

***Authors’ response:*** We acknowledge reviewer concern. We do plan to use JBI tool to assess the quality of the included papers and thus revise the section accordingly. In addition, only cross-sectional studies will be included as they are the most appropriate design for estimating prevalence and associated factors in the target population **(Line 168-170).** In the data management section, we have revised the line for better clarification **(p8, Line 188).**

6. **Reviewer’s Comment:** Data management

• Do you mean Data selection?

***Authors’ response:*** Yes, and the section heading has been revised accordingly.

7. **Reviewer’s Comment:** Data extraction

• Line 219: pretested or structured? which one do you thick better?

• Line 221: what if not agree?

***Authors’ response:*** In previous Line 219 we think pre-tested would be perfect to use and revised accordingly. We have clarified in the revised version that if the independent reviewer were not agreed, third reviewer will intervein and give a solution furthermore.

8. **Reviewer’s Comment:** Data synthesis and statistical analysis:

• Line 240: what other methods?

• Line 254: if the number of studies are >=10

• Line 2540: For what do you plan to perform sensitivity analysis

• Line 261: what about subgroup analysis and publication bias analysis?

***Authors’ response:*** In previous line 240 we have added other methods like random-effect model and complemented by subgroup and sensitivity analysis to explore sources of heterogeneity. In previous line 254 we have added the reviewer suggested line accordingly. We have also clarified the reason for performing sensitivity analysis to find if any single study effect on the pooled estimated results or not. Lastly, we do add our plan to have subgroup and publication bias analysis (Funnel plot and egger’s test) in the revised manuscript accordingly **(p9, Line 212-244).**

9. **Reviewer’s Comment:** Potential Impact

Line 318 not necessary at this stage. you will address it latter

***Authors’ response:*** The suggestion has been addressed accordingly in the revised version and the line has been removed.

Thank you for your kind consideration.
